# Overweight and obesity in adult patients with phenylketonuria: a systematic review

**DOI:** 10.1186/s13023-023-02636-2

**Published:** 2023-02-22

**Authors:** Aurel T. Tankeu, Despina Christina Pavlidou, Andrea Superti-Furga, Karim Gariani, Christel Tran

**Affiliations:** 1 Division of Genetic Medicine, University of Lausanne and University Hospital of Lausanne, Lausanne, Switzerland; 2 Service of Endocrinology, Diabetes, Nutrition and Therapeutic Patient Education, Geneva University Hospitals and Geneva University, Geneva, Switzerland

**Keywords:** Phenylketonuria, Obesity, Overweight, Adult

## Abstract

**Background:**

Excess weight is a rising concern in patients with phenylketonuria (PKU). It is commonly observed in children and adolescents with PKU; but data on adults are inconsistent. This review aims to summarize available data on excess weight in adult PKU individuals.

**Methods:**

We conducted a systematic search of literature in English, from inception to October 2021, on PubMed and Embase to identify articles on overweight and obesity in adult PKU patients. Prevalence of overweight and obesity, body mass index (BMI) and gender differences were the outcomes of interest.

**Results:**

Of 260 articles identified, only 8 fulfilled quality criteria for inclusion after screening of titles, abstracts and full texts. The mean BMI of adult PKU patients in these studies ranged from 26 ± 5.4 to 30.3 ± 1.8 kg/m^2^. When compared to matched controls, adult PKU patients had higher BMI and higher prevalence of obesity. However, results were inconsistent when PKU adults were compared to the general population. The prevalence of obesity in the included studies varied widely between 4.5% up to 72% in individual studies. Obesity was 2–3 times more frequent in female PKU patients.

**Conclusions:**

Excess weight is frequent in adult PKU patients, especially in females, even if the difference with the general population is debatable. The heterogeneity of the studies makes it difficult to interpret the results and the factors that contribute to obesity. Content of the diet, psychological status, diet-associated disordered eating, patient’s social environment and lifestyle are listed as potentials contributors to excess weight in PKU adult population. Further studies are needed to better elucidate this question. In the meantime, weight control and healthy eating habits should be considered in the management and follow-up of these patients.

**Supplementary Information:**

The online version contains supplementary material available at 10.1186/s13023-023-02636-2.

## Background

Phenylketonuria (PKU; OMIM#261600) is a genetic metabolic disease caused by the deficiency of phenylalanine hydroxylase, a liver enzyme responsible for the conversion of phenylalanine to tyrosine [1]. When untreated, phenylalanine hydroxylase deficiency results in high phenylalanine blood levels that can cross the blood–brain barrier and impair brain development resulting in intellectual disability, microcephaly, autism, seizures, and psychiatric disorders [2]. First described by Følling in 1934, in 1953 PKU became the first inherited error of metabolism for which an effective treatment was found [3, 4]. Dietary phenylalanine restriction was found to result in a marked reduction in blood phenylalanine levels leading to a remarkable improvement in behavior and development of affected children [1, 3]. Since then, the phenylalanine-restricted diet supplemented with phenylalanine-free amino acid formula and special low-protein foods represents the mainstay of PKU treatment [1, 2]. The implementation of this dietary treatment along with the instauration of newborn screening program (NBS) in many countries allowed early diagnosis and thus prevention or attenuation of intellectual disability in most patients [5, 6]. Seventy years later, while following the first generations of PKU adult patients who benefited from early treatment and normal development free from neurological complications, we are confronted with new medical challenges. Weight control appears as an emerging concern in PKU individuals with an increasing amount of publications reporting excess weight in children as well as in adult patients with PKU. A recent systematic review by Sena et al. [7], concluded that excess weight is common in children and adolescents with PKU. However, data in adults are inconsistent. For instance, Azabdaftari et al., reported a significantly higher body mass index (BMI) in PKU patients compared to healthy controls while Robertson et al. found similar percentage of overweight and obesity in PKU patients compared to the general population [8, 9]. In this situation, adult metabolic clinics struggle with an unmet need in the management of PKU patients presenting with overweight and obesity: how could weight control be combined with a strict protein-restricted diet? This systematic review aims to summarize available evidence on overweight and obesity in adult individuals with PKU. The relevant data could help to provide baseline information for the effective management of both PKU and excess weight.

## Methods

### Data source and search strategy

This systematic review was conducted according to Preferred Reporting Items for Systematic reviews and Meta-Analysis (PRISMA) guideline [10]. We conducted a literature search on PubMed and Excerpta Medica Database (Embase) in order to identify relevant abstracts published from inception to October 2021, reporting on the prevalence of overweight and/or obesity in PKU individuals or their BMI. The keywords used for the search included ‘phenylketonuria', ‘obesity’ or ‘overweight’. In addition, the references list of all selected studies were also reviewed as complement of the bibliographic search (Additional file 1: Table S1).

### Study selection

We selected observational studies (cross-sectional, case–control or cohort studies) including individuals with PKU with or without control group and reporting at least one of the relevant parameter (body weight, mean/median BMI, frequency of overweight and/or obese patients). Three authors (AT, DP & KG) independently and blindly screened the titles and abstracts of selected articles, retrieved from the literature for eligibility using Rayyan app for systematic reviews [11]. Then, the same authors downloaded and assessed full texts of eligible articles for inclusion. A fourth author (CT) resolved any disagreement during this process. The study protocol is accessible for review online in the NIHR International Prospective Register of Systematic Reviews (PROSPERO: ID = CRD42020177688).

### Data extraction and risk of bias assessment

We used a preconceived data form to collect information of selected articles. Two authors (AT & DP) extracted relevant data from each included article such as year of publication, study’s location and design, sample size, gender, age, PKU type, diet, body weight, BMI, frequency of overweight and obesity. Overweight and obesity were considered as BMI ≥ 25 kg/m^2^ and a BMI ≥ 30 kg/m^2^, respectively [12]. Additionally, the same authors using the NIH’s Quality Assessment Tool for Observational Cohort and Cross-Sectional Studies, independently assessed studies’ quality [13]. Studies were assigned an answer (no, yes, other) to each of the 14 questions of the assessment tool. Studies were then classified as good, fair or poor by each author (Additional file 1: Table S2) based on the overall assessment of the author.

## Results

### Studies’ characteristics

**Fig. 1:**
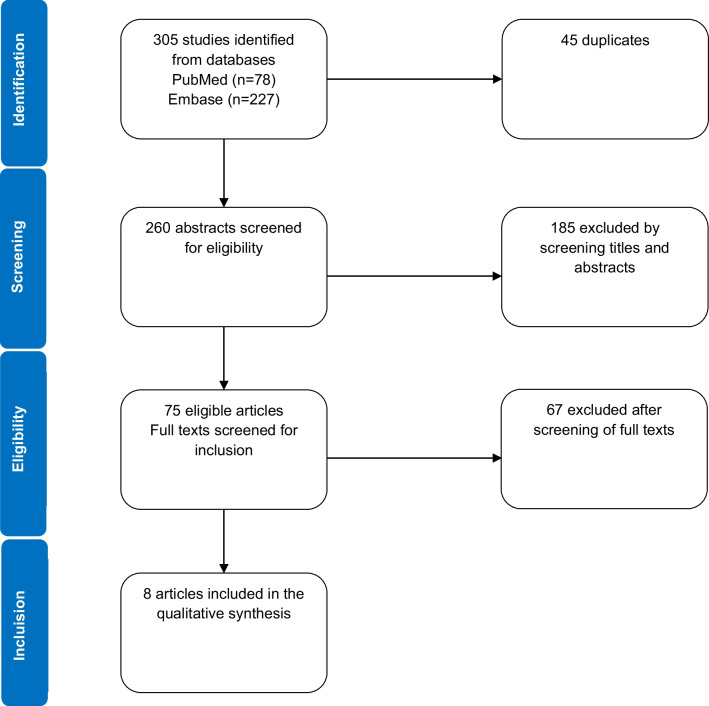
Study selection flow chart

The study’s flow chart is depicted in Fig. 1. Through systematic search in databases, we identified 305 potential articles, 45 of which were duplicates. From the remaining 260 articles, after screening by titles and abstracts, we selected 75 eligible articles for full text screening; the other studies were discarded because they did not fulfill eligibility criteria, and only eight studies were included in the review. All articles included were published between 2013 and 2019. These studies reported either BMI, overweight and/or obesity prevalence in adult PKU patients. The studies included and their main features are described in Tables 1 and 2. Among the eight studies included in the review, three studies had a control group for comparison including PKU patients matched to healthy individuals on ratios 1:1, 1:5, and 1:10 [8, 14, 15]. Three studies compared BMI, overweight and/or obesity rates of PKU patients to the general population [9, 16, 17]. One study did not compare PKU patients to matched controls or general population, but reported both BMI and obesity frequencies [18]. A second study reported PKU patients to controls but with a ratio lower than 1:1 [19]. There was some heterogeneity between studies regarding the diagnosis context, and type of PKU. For instance, four studies did not specify the diagnosis context of PKU (NBS, family screening or clinical symptoms) [8, 14, 16, 18]. Two studies reported patients diagnosed through NBS [9, 17] and in the two others, patients were diagnosed through NBS and clinical suspicion [15, 19]. Similarly, one study included patients with different phenotypes of PKU (classic, mild, BH4 responsive or even hyperphenylalaninemia) [8]. Additionally, the treatment of PKU was different depending on studies. Three studies included adult patients only on phenylalanine restricted-diet [8, 9, 18] while four studies included patients with both diet and/or pharmacological treatment (sapropterin dihydrochloride) [15–17, 19]. Finally, the degree of metabolic control, as indicated by the plasma phenylalanine levels, was variable between studies.

**Table 1:** Studies and patients’ characteristics

First author, year	Design	Location	Funding sources	Matching criteria	Sample size		Treatment	Diagnosis context	Mean/median age (years)	
					PKU	Controls	PKU		PKU	Controls
Azabdaftari, 2019 [8]	Cross-sectional	Germany	Funds dedicated to clinical research	Age	23	28	Diet	NR	30.8 ± 8.4	30.1 ± 9.1
Burton, 2018 [14]	Retrospective	USA	Pharmaceutical company	Age, race, gender, geographic location	3691	18455	NR	NR	34.6 ± 14.3	34.9 ± 14.2
Trefz, 2019 [15]	Retrospective	Germany	Pharmaceutical company	Age and gender	377	3770	Diet + pharmacological treatment	NBS/clinical suspicion	50.9 ± 20.4	NR
Robertson, 2013 [9]	Retrospective	UK	None	NA	236	NA	Diet	NBS	26 ± 7	NA
Jani, 2017 [16]	Longitudinal	USA	NIH and Pharmaceutical company	NA	27	NA	Diet + pharmacological treatment	NR	28.8 (19.5, 54.6)	NA
Ozel, 2014 [17]	Retrospective and cross-sectional	Europe and Turkey	None	NA	164	NA	Diet + pharmacological treatment	NBS	22.9(Ankara) 19.6(Brussels)	NA
Couce, 2018 # [19]	Cross-sectional	Spain	APS fees covered by NPO	Age and gender	41	25	Diet + pharmacological treatment	NBS/clinical suspicion	NR	NR
Williams, 2015 [18]	Cross-sectional	Australia	None	NA	41	NA	Diet	NR	31 ± 12	NA

*APS* article processing charges, *NA* not applicable, *NBS* newborn screening, *NIH* National Institute of Health, *NPO* Non-profitable Organization, *NR* not reported, *PKU* phenylketonuria, *UK* United Kingdom, *USA* United States of America

^#^Data are extrapolated from original data of the study

**Table 2:** Excess weight reported in individual studies

First author, year	Sample size		PKU gender		Mean/median BMI (kg/m^2^)		Overweight frequency (%)		Obesity frequency (%)		PR (95% CI)	Trend (*p* value)
	PKU	Controls	Males	Females	PKU	Controls	PKU	Controls	PKU (female/male)	Controls		
Azabdaftari, 2019 [8]	23	28	13	10	27.6 ± 5.4	23.4 ± 6.4	19	NR	33 (50/18)	NR	NA	*p* < 0.001
Burton, 2018 [14]	3691	18455	1341	2350	NR	NR	5.35	2.25	NR	NR	2.06	*p* < 0.0001
Trefz, 2019 [15]	377	3770	158	219	NR	NR	15.9*	11.2*	*	*	1.43 (1.11–1.83)	Signifcantly different
Robertson, 2013 [9]	236	NA	115	121	26 ± 5.4	NA	31	NA	24 (34/17)	NA	NA	Similar to background population
Jani, 2017 [16]	27	NA	9	18	27.1	NA	NR	NA	NR	NA	NA	Slightly higher than background population
Ozel, 2014 [17]	164	NA	63	101	NR	NA	15.2–42	NA	4.5–24.2	NA	NA	Less or similar to background population
Couce,# 2018 [19]	41^#^	25^#^	17^#^	24^#^	NR	NR	35.9^#^	12.9^#^	17.9^#^	22.5^#^	NA	NA
Williams, 2015 [18]	41	NA	15	26	30.3 ± 1.8	NA	14	NA	72	NA	NA	NA

*CI* confidence interval, *NA* not applicable, *NR* not reported, *PKU* phenylketonuria, *PR* prevalence ratio

*Total prevalence of overweight and obesity (BMI of ≥ 25 kg/m^2^)

^#^Data are extrapolated from original data of the study

### Overweight and obesity in PKU individuals

Table 1 summarizes patients’ features and outcomes in the studies included. Overall, BMI of adult PKU patients in studies included varied between 26 ± 5.4 kg/m^2^ and 30.3 ± 1.8 kg/m^2^. The prevalence of obesity varied from 4.5% up to 72%. The first three studies presented in Table 1 are studies comprising a control group [8, 14, 15]. These studies found higher BMI and obesity prevalence in adults with PKU compared to controls. Specifically, Burton et al. [14] reported significantly higher prevalence of obesity in PKU individuals compared to matched controls (5.35% vs. 2.25%, *p* < 0.0001). Similarly, Azabdaftari et al. [8] found that adult PKU patients have significantly higher BMI than controls (27.6 ± 5.4 kg/m^2^ vs. 23.4 ± 6.4 kg/m^2^, *p* < 0.001). These authors also reported a prevalence of 19% and 33% for overweight and obesity, respectively. In the same vein, Trefz et al. [15] reported significantly higher prevalence of overweight and obesity in early diagnosed adult PKU patients compared to controls (11.8% vs. 7.1%, PR:1.67). They also reported similar data for their whole study population with higher prevalence of overweight and obesity in PKU adults compared to controls (15.9% vs. 11.2%, PR:1.43).

On the other hand, three studies included in the review did not have a control group but compared data of their adult PKU patients to the background population. In this context, Jani et al. [16] reported a higher median BMI for adult male (28.5 kg/m^2^ vs. 27.7 kg/m^2^) and female (30.5 kg/m^2^ vs. 27.7 kg/m^2^) PKU patients compared to their respective general population. Conversely, in the study in the United Kingdom by Robertson et al. [9], 31% and 24% of PKU individuals were overweight and obese respectively, but these figures were similar to those of the background United Kingdom population. In a multicenter study from 6 countries, Ozel et al. found that the majority of the centers (4 out of 6) had similar or lower rates of obesity in PKU when compared to the respective background populations. Overweight was less frequent in adult PKU patients for 5 out of 6 centers when compared to the general population of interest [17]. Couce et al. [19] studied a PKU population aged between 4 and 52 years. Extrapolated data from this studies showed that 35.9% of adult patients were overweight and 17.9% were obese. Williams et al. [18] reported a mean BMI of 30.3 ± 1.8 kg/m^2^ in adult PKU patients with up to 72% of obese and 14% overweight PKU patients.

### Gender differences in overweight and obesity of PKU individuals

Four out of eight studies included in this review provided data regarding males vs females prevalence of obesity or overweight [8, 9, 16, 17]. Azabdaftari et al. [8] reported that 50% of female PKU patients were obese vs 18% of male patients. Robertson et al. [9] reported that the frequency of obesity was twice higher in females than males (34% and 17%, respectively). In their multicenter study, Ozel et al. [17] found higher rates of obesity in females compared to males in 5 of 6 centers with a median prevalence of 20% in females versus 7% in males. Finally, Jani et al. [16] reported a median BMI of 30.5 kg/m^2^ in adult PKU females vs 28.5 kg/m^2^ in males.

## Discussion

From discovery to treatment, PKU is a medical success story. The effectiveness of the screening program worldwide and the success of the dietary treatment have enabled tens of thousands of people who might have suffered from severe intellectual disability to lead almost normal lives [20, 21]. However, in the mid-70s, the discovery of maternal PKU fetopathy in the children of patients whose diet had been liberalized led to the conclusion that “we have changed the natural history of the disease and consequently recognized new sequelae” [21]. Today, as we observe the first generation of PKU patients treated since birth, we continue to learn and wonder whether by actively treating these patients with a highly restrictive and modified diet, we may contribute to other metabolic disorders such as obesity.

Holm et al. [22] and White et al. [23] were the first to draw attention to the issue of excess weight in PKU. Using the data from the PKU Collaborative Study in 1982, they both reported that children with PKU tended to have higher BMI than their controls and were overweight on average by age 4 [22, 23]. This observation was confirmed four decades later in a systematic review, which concluded that “excess weight was a common outcome in children and adolescents with PKU” [7]. However, the data for adults are less conclusive, probably also due to the under-representation of adults in the available studies. Given that, we aimed to summarize available data from studies focusing on adult patients.

Our systematic review of the literature showed that the prevalence of obesity in adult PKU patients was quite variable between studies, ranging from a minimum of 4.5% to a maximum of 72%. In studies were PKU patients were compared to matched controls, BMI of PKU individuals was higher and varied from 26 ± 5.4 kg/m^2^ to 30.3 ± 1.8 kg/m^2^. Adult PKU patients also had higher prevalence of obesity thus supporting the notion of excess weight in this group. Surprisingly, however, studies comparing PKU adults to the general population have given inconsistent results: some studies have found higher rates of obesity in PKU, other studies have found lower rates of obesity, and some studies found no difference between PKU adults and the background population. The reasons for these discrepancies are not clear; it seems likely that these inconsistencies have their origins in the ascertainment of matched controls and/or in the assessment of the general population.

Our findings are supported also by the recent publication of Dios-Fuentes et al. [24] including an adult cohort of 90 PKU patients in Spain. PKU patients participating in this study had a median BMI of 26.61 kg/m^2^ (22.7–31.1 kg/m^2^) and 29.2% were obese. This is slightly higher than the prevalence in the background population estimated to 23.8% [25]. However, a recent systematic review and meta-analysis of 12 studies by Rodrigues et al. [26] found no differences in BMI between PKU patients and healthy controls. We speculate that the differences between our findings and those of Rodrigues et al. might be due to the different populations included in each review, given that Rodrigues et al. included both pediatric and adult populations while our review focused only on adults. Children and/or pre-adolescents included in the review from Rodrigues et al. are supposedly different from adolescents and adults, not only in terms of physiology, but also in terms of food-related behavior. These differences could contribute to the conflicting results found by the two reviews. For illustration, Sena et al. [7], in their review on overweight and obesity in children and adolescents, found a higher prevalence of overweight compared to the reference population when analyzing older age groups (near adolescence) supporting our findings.

Our tentative conclusion is that on the background of a tendency to overweight in children with PKU, adults with PKU seem to follow a similar trend to overweight and obesity but methodological issues do not allow a solid statement yet; more studies are needed. A tendency to overweight in individuals with PKU (children, adolescents and potentially adults) is certainly problematic. One might expect that the dietary monitoring required in PKU treatment might offer the opportunity of tailoring the diet and thus to prevent overweight and obesity. Yet, the opposite seems to be the case. Why is this the case? This remains unclear, and potential explanations are manifold.

The most frequently invocated mechanism is that of a diet-associated (our even diet-induced) disordered eating [27–32]. Rigid dietary control with low flexibility regarding the timing, content, amount of foods and dichotomous thinking presenting food (or some foods) as good or bad as imposed in PKU patients is associated with increased frequency of disordered eating [29]. Additionally, disordered eating have been reported in many chronic conditions requiring a lifelong diet, with pressure to maintain a strict restricted diet as well as feelings of social isolation due to dietary restriction [32, 33]. Therefore, adults with PKU, whose diet was controlled in terms of quantity and type of food with an ongoing concern about phenylalanine levels, may be at particular risk of developing eating disorders [29]. Simply put, individuals with PKU might tend to compensate the restriction of protein-rich food by overeating those foods that are not restricted.

Another hypothesis suggests that excess weight in adult patients might be related to the composition of the diet itself. Carbohydrate intake is probably higher in PKU individuals due to their specific diet. Some authors claimed that low-phenylalanine containing foods are rich in carbohydrates [34–36]. For instance, Pena et al. [35] in their analysis of these special low-protein foods in nine countries, reported that 75% of these products have higher energy content than regular foods. Similarly, Woods et al., showed that 68% of special low-protein foods contained more carbohydrates than their equivalents with normal protein contain. In addition, carbohydrate sources with low fiber content and high glycemic index as found in these products are known as causative factor for overweight and obesity [37].

A third factor contributing to overweight in PKU might be reduced physical activity. There is limited evidence regarding physical activity levels in adult patients with PKU. Some authors have suggested that social isolation, mood alteration, lack of specific recommendations regarding physical activity, together with low incentive to practice physical activities as well as disease-related stress, might contribute to a low physical activity in this population [26, 37]. On the other hand, Mazzola et al. [38] reported similar physical activity level and fitness parameters in PKU patients compared to controls. In the study of Jani et al. [16] using a self-reported evaluation with the IPAQ (international physical activity questionnaire), all patients with PKU reported being physically active with 85% and 54% reporting light to moderate physical activity levels suggesting that sedentary lifestyle might not be a major factor in the development of obesity in this population. Others factors such as socio-economic status and intelligence quotient scores might also be involved but are yet to be evaluated in patients with PKU. More recently, a homozygous *Pah-R261Q* mouse model was generated, the R261Q variant being one of the most abundant among PKU patients. It revealed unexpected traits, including higher body weight in males, altered lipid metabolism and a metabolic profile indicative of oxidative stress suggesting a possible contribution of the genotype to comorbid associations reported in PKU [39].

Interestingly, our review also indicated that obesity appeared to be two to three times more frequent in women than men with PKU. These results are similar to previous finding by Sena et al. [7] in the pediatric population where they reported a significantly higher occurrence of obesity in girls with PKU aged between 8 and 18 years old as well as an increase in body fat mass in adolescent females compared with the healthy population. The reasons behind the high frequency of excess weight and obesity in females with PKU remain unclear but might be related to the reportedly higher frequency of eating disorders in females [40]. A few studies have addressed the prevalence of disordered eating in PKU patients [41, 42], and the European Guidelines for PKU as well as a recently published international consensus contain explicit warnings about the possible occurrence of eating disorders [43, 44]. However, data on eating disorders in PKU adults are scarce [45] and deserve to be explored better [2]. It may also be noted that women with PKU are more likely to remain in regular contact with their metabolic center because of the need for strict dietary treatment during pregnancy and, therefore, the data collected on BMI may be available for more female patients than male patients. Future studies specifically examining the effect of gender on obesity in patients with PKU should help answer this question.

It is important to consider the limitations of our review. The main limitation is the heterogeneity of the studies included. Studies were heterogeneous regarding the sample size, study design and risk of assessment bias. Some studies have a control group and compared data of adult PKU patients with healthy matched individuals while other authors compared data of their adult patients with the background population without any matching. Given that overweight and obesity can often relate to social and environmental factors, matching controls on age and gender only may not reflect differences in environment or learning eating habits, which would possibly be risk factors in the development of excess weight. There was also significant heterogeneity among participants included in the studies [i.e. context of diagnosis (NBS, non-NBS), metabolic control, pharmacological and concurrent dietary treatment]. It is therefore unclear whether current dietary and/or pharmacological treatment in adulthood may be contributing to overweight and obesity, or if the nature of dietary treatment starting from early infancy leads to eating behaviors that increase the risk of excess weight, potentially independently from adult therapy. Better quality and more homogenous studies are needed in order to clarify which patients are more prone to develop overweight or obesity. In addition, all the studies included in this review used BMI as a single measure of obesity. However, using only BMI to assess obesity might not give the complete picture. Therefore, it might be important for future studies in the field to consider more than one method to assess obesity including body composition for example. Finally, we did not discuss environmental and geographical differences in obesity prevalence that are beyond the scope of this review given that all the included studies were carried out in the developed countries that present similar obesogenic environments.

## Conclusions

This literature review suggests that in spite of inconsistencies between studies that we attribute to methodological issues, adult patients with PKU are at increased risk of overweight and obesity when compared to matched controls. Excess weight seems to develop during childhood and is carried out through adolescence leading to obesity in adulthood. In view of these observations, we suggest that while a good control of phenylalanine levels in PKU patients remains the top priority, attention should be given that this is not obtained at the expense of excessive weight gain. It is likely that upstream work from childhood onwards, taking into account the patient's social environment, psychological status, relationship to food and lifestyle, will make it possible to identify risk factors on which to act to prevent obesity. For PKU patients presenting with obesity, a multidisciplinary team involving the metabolic team and obesity clinics for adapted nutritional counseling, personalized exercise and psychological support might facilitate and improve the management.

## Supplementary Information

**Additional file 1: Table 1.** Search strategy for each database.** Table 2.** Risk of bias assessment for each included study

## Data Availability

The datasets used and/or analyzed during the current study are available from the corresponding author (CT) on reasonable request.

## Abbreviations

BH4: Tetrahydrobiopterin (sapropterin)
BMI: Body mass index
NBS: Newborn screening
NIH: National Institute of Health
NIHR: National Institute for Health and Care Research
OMIM: Online Mendelian Inheritance in Man
PKU: Phenylketonuria
Vs: Versus

## References

[1] Blau N, van Spronsen FJ, Levy HL (2010). Phenylketonuria. The Lancet.

[2] MacDonald A, van Wegberg AMJ, Ahring K, Beblo S, Belanger-Quintana A, Burlina A (2020). PKU dietary handbook to accompany PKU guidelines. Orphanet J Rare Dis.

[3] Bickel H, Gerrard J, Hickmans E (1953). Preliminary communication. The Lancet.

[4] Folling I (1994). The discovery of phenylketonuria. Acta Paediatr Suppl.

[5] Groselj U, Tansek MZ, Battelino T (2014). Fifty years of phenylketonuria newborn screening—a great success for many, but what about the rest?. Mol Genet Metab.

[6] Guthrie R, Susi A (1963). A simple phenylalanine method for detecting phenylketonuria in large populations of newborn infants. Pediatrics.

[7] Sena BDS, Andrade MIS, Silva A, Dourado KF, Silva ALF (2020). Overweight and associated factors in children and adolescents with phenylketonuria: a systematic review. Rev Paul Pediatr.

[8] Azabdaftari A, van der Giet M, Schuchardt M, Hennermann JB, Plockinger U, Querfeld U (2019). The cardiovascular phenotype of adult patients with phenylketonuria. Orphanet J Rare Dis.

[9] Robertson LV, McStravick N, Ripley S, Weetch E, Donald S, Adam S (2013). Body mass index in adult patients with diet-treated phenylketonuria. J Hum Nutr Diet.

[10] Moher D, Liberati A, Tetzlaff J, Altman DG, Group P (2009). Preferred reporting items for systematic reviews and meta-analyses: the PRISMA statement. BMJ.

[11] Ouzzani M, Hammady H, Fedorowicz Z, Elmagarmid A (2016). Rayyan-a web and mobile app for systematic reviews. Syst Rev.

[12] Chooi YC, Ding C, Magkos F (2019). The epidemiology of obesity. Metabolism.

[13] National Heart L, and Blood Institute. Quality Assessment Tool for Observational Cohort and Cross-Sectional Studies 2013. https://www.nhlbi.nih.gov/health-topics/study-quality-assessment-tools.

[14] Burton BK, Jones KB, Cederbaum S, Rohr F, Waisbren S, Irwin DE (2018). Prevalence of comorbid conditions among adult patients diagnosed with phenylketonuria. Mol Genet Metab.

[15] Trefz KF, Muntau AC, Kohlscheen KM, Altevers J, Jacob C, Braun S (2019). Clinical burden of illness in patients with phenylketonuria (PKU) and associated comorbidities—a retrospective study of German health insurance claims data. Orphanet J Rare Dis.

[16] Jani R, Coakley K, Douglas T, Singh R (2017). Protein intake and physical activity are associated with body composition in individuals with phenylalanine hydroxylase deficiency. Mol Genet Metab.

[17] Gokmen Ozel H, Ahring K, Belanger-Quintana A, Dokoupil K, Lammardo AM, Robert M (2014). Overweight and obesity in PKU: the results from 8 centres in Europe and Turkey. Mol Genet Metab Rep.

[18] Williams RA, Hooper AJ, Bell DA, Mamotte CD, Burnett JR (2015). Plasma cholesterol in adults with phenylketonuria. Pathology.

[19] Couce ML, Sanchez-Pintos P, Vitoria I, De Castro MJ, Aldamiz-Echevarria L, Correcher P (2018). Carbohydrate status in patients with phenylketonuria. Orphanet J Rare Dis.

[20] Brosco JP, Paul DB (2013). The political history of PKU: reflections on 50 years of newborn screening. Pediatrics.

[21] Green A (2020). Sheila: unlocking the treatment for PKU.

[22] Holm VA, Kronmal RA, Williamson M, Roche AF (1979). Physical growth in phenylketonuria: II. Growth of treated children in the PKU collaborative study from birth to 4 years of age. Pediatrics.

[23] White JE, Kronmal RA, Acosta PB (1982). Excess weight among children with phenylketonuria. J Am Coll Nutr.

[24] Dios-Fuentes E, Gonzalo Marin M, Remon-Ruiz P, Benitez Avila R, Bueno Delgado MA, Blasco Alonso J (2022). Cardiometabolic and nutritional morbidities of a large, adult, PKU cohort from Andalusia. Nutrients.

[25] Review WP. Obesity rates by countries. 2022.

[26] Rodrigues C, Pinto A, Faria A, Teixeira D, van Wegberg AMJ, Ahring K (2021). Is the phenylalanine-restricted diet a risk factor for overweight or obesity in patients with phenylketonuria (PKU)? A systematic review and meta-analysis. Nutrients.

[27] Barrack MT, West J, Christopher M, Pham-Vera AM (2019). Disordered eating among a diverse sample of first-year college students. J Am Coll Nutr.

[28] Haitjema S, Lubout CMA, Abeln D, Bruijn-van der Veen M, MacDonald A, Wolffenbuttel BHR (2022). Dietary treatment in Dutch children with phenylketonuria: an inventory of associated social restrictions and eating problems. Nutrition.

[29] Linardon J, Mitchell S (2017). Rigid dietary control, flexible dietary control, and intuitive eating: evidence for their differential relationship to disordered eating and body image concerns. Eat Behav.

[30] McCuen-Wurst C, Ruggieri M, Allison KC (2018). Disordered eating and obesity: associations between binge-eating disorder, night-eating syndrome, and weight-related comorbidities. Ann N Y Acad Sci.

[31] Polivy J, Herman CP (2002). Causes of eating disorders. Annu Rev Psychol.

[32] Quick VM, Byrd-Bredbenner C, Neumark-Sztainer D (2013). Chronic illness and disordered eating: a discussion of the literature. Adv Nutr.

[33] Luu S, Breunig T, Drilias N, Kuhl A, Scott Schwoerer J, Cody P (2020). A Survey of eating attitudes and behaviors in adolescents and adults with phenylalanine hydroxylase deficiency. WMJ.

[34] Moretti F, Pellegrini N, Salvatici E, Rovelli V, Banderali G, Radaelli G (2017). Dietary glycemic index, glycemic load and metabolic profile in children with phenylketonuria. Nutr Metab Cardiovasc Dis.

[35] Pena MJ, Almeida MF, van Dam E, Ahring K, Belanger-Quintana A, Dokoupil K (2015). Special low protein foods for phenylketonuria: availability in Europe and an examination of their nutritional profile. Orphanet J Rare Dis.

[36] Wood G, Evans S, Pointon-Bell K, Rocha JC, MacDonald A (2020). Special low protein foods in the UK: an examination of their macronutrient composition in comparison to regular foods. Nutrients.

[37] Rocha JC, MacDonald A, Trefz F (2013). Is overweight an issue in phenylketonuria?. Mol Genet Metab.

[38] Mazzola PN, Teixeira BC, Schirmbeck GH, Reischak-Oliveira A, Derks TGJ, van Spronsen FJ (2015). Acute exercise in treated phenylketonuria patients: physical activity and biochemical response. Mol Genet Metab Rep.

[39] Aubi O, Prestegard KS, Jung-Kc K, Shi TS, Ying M, Grindheim AK (2021). The Pah-R261Q mouse reveals oxidative stress associated with amyloid-like hepatic aggregation of mutant phenylalanine hydroxylase. Nat Commun.

[40] Sangvai D (2016). Eating disorders in the primary care setting. Prim Care.

[41] Bilder DA, Kobori JA, Cohen-Pfeffer JL, Johnson EM, Jurecki ER, Grant ML (2017). Neuropsychiatric comorbidities in adults with phenylketonuria: a retrospective cohort study. Mol Genet Metab.

[42] Luu S, Breunig T, Drilias N, Kuhl A, Schwoerer SJ, Cody P (2020). A survey of eating attitudes and behaviors in adolescents and adults with phenylalanine hydroxylase deficiency. WMJ Mars.

[43] Burton BK, Hermida A, Belanger-Quintana A, Bell H, Bjoraker KJ, Christ SE (2022). Management of early treated adolescents and young adults with phenylketonuria: development of international consensus recommendations using a modified Delphi approach. Mol Genet Metab.

[44] van Wegberg AMJ, MacDonald A, Ahring K, Belanger-Quintana A, Blau N, Bosch AM (2017). The complete European guidelines on phenylketonuria: diagnosis and treatment. Orphanet J Rare Dis.

[45] Antisdel JE, Chrisler JC (2000). Comparison of eating attitudes and behaviors among adolescent and young women with type 1 diabetes mellitus and phenylketonuria. J Dev Behav Pediatr.

