# Supplementary material for: Overweight and obesity in adult patients with phenylketonuria: a systematic review
Source: Orphanet J Rare Dis. 2023 Feb 22;18:37. doi: 10.1186/s13023-023-02636-2 (PMC9945382; doi:10.1186/s13023-023-02636-2)
Supplement: Supplementary file 1 — Additional file 1: Table 1. Search strategy for each database. Table 2. Risk of bias assessment for each included study [file 13023_2023_2636_MOESM1_ESM.docx]

**Supplementary Table 1: Search strategy for each database**

|  | **Search terms in Embase** |  |
| --- | --- | --- |
| # 1 | 'phenylketonuria'/exp OR phenylketonuria |  |
| # 2 | 'obesity'/exp OR obesity OR 'overweight'/exp |  |
| # 3 | # 1 AND # 2 |  |
| # 4 | Limits from inception to October 31, 2021 | 227 hits |
|  | **Search terms in PubMed** |  |
| # 1 | "phenylketonurias"[MeSH Terms] OR "phenylketonurias"[All Fields] OR  "phenylketonuria"[All Fields] |  |
| # 2 | "obeses"[All Fields] OR "obesity"[MeSH Terms] OR "obesity"[All Fields] OR "obese"[All Fields] OR "obesities"[All Fields] OR "obesity's"[All Fields] |  |
| # 3 | # 1 AND # 2 |  |
| # 4 | Limits from inception to October 31, 2021 | 78 hits |

| **First author/year** | **Q1** | **Q2** | **Q3** | **Q4** | **Q5** | **Q6** | **Q7** | **Q8** | **Q9** | **Q10** | **Q11** | **Q12** | **Q13** | **Q14** | **Risk of bias** |
| --- | --- | --- | --- | --- | --- | --- | --- | --- | --- | --- | --- | --- | --- | --- | --- |
| Williams, 2015 (18) | + | + | ? | + | -- | + | ? | -- | + | -- | + | -- | ? | -- | Moderate |
| Azabdaftari, 2019 (8) | + | + | + | + | + | + | ? | -- | + | -- | + | -- | + | + | Low |
| Trefz, 2019 (15) | + | + | + | + | -- | + | ? | -- | + | -- | + | -- | ? | -- | Moderate |
| Robertson, 2013 (9) | + | + | ? | + | -- | + | ? | + | + | + | + | -- | ? | -- | Moderate |
| Burton, 2018 (14) | + | + | + | + | -- | + | ? | -- | + | -- | + | -- | ? | + | Moderate |
| Jani, 2017 (16) | + | + | ? | + | -- | + | ? | -- | + | -- | + | -- | ? | + | Moderate |
| Ozel, 2014 (17) | + | + | ? | + | -- | + | ? | -- | + | -- | -- | -- | ? | -- | Low |
| Couce, 2018 (19) | + | + | ? | + | -- | + | ? | + | + | -- | + | -- | ? | + | Moderate |

**Supplementary Table 2: Risk of bias assessment for each included study**
